# Low-dose calcipotriol can elicit wound closure, anti-microbial, and anti-neoplastic effects in epidermolysis bullosa keratinocytes

**DOI:** 10.1038/s41598-018-31823-6

**Published:** 2018-09-07

**Authors:** Christina Guttmann-Gruber, Birgit Tockner, Cornelia Scharler, Clemens Hüttner, John E. Common, Angeline S. L. Tay, Simon L. I. J. Denil, Alfred Klausegger, Andrea Trost, Jenny Breitenbach, Peter Schnitzhofer, Peter Hofbauer, Martin Wolkersdorfer, Anja Diem, Martin Laimer, Dirk Strunk, Johann W. Bauer, Julia Reichelt, Roland Lang, Josefina Piñón Hofbauer

**Affiliations:** 1 EB House Austria, Research Program for Molecular Therapy of Genodermatoses, Department of Dermatology, University Hospital of the Paracelsus Medical University Salzburg, Salzburg, Austria; 2 Experimental & Clinical Cell Therapy Institute, Spinal Cord Injury and Tissue Regeneration Center Salzburg (SCI-TReCS), PMU Salzburg, Salzburg, Austria; 3 Institute of Medical Biology, A*STAR, 8A Biomedical Grove, Immunos #06-08, Singapore, Singapore; 4 Skin Research Institute of Singapore, A*STAR, 8A Biomedical Grove, Immunos #06-06, Singapore, Singapore; 5 University Clinic of Ophthalmology and Optometry, Research Program for Ophthalmology and Glaucoma Research, Paracelsus Medical University Salzburg, Salzburg, Austria; 6 Landesapotheke Salzburg, Department of Production, Hospital Pharmacy, Salzburg, Austria; 7 Department of Dermatology, University Hospital of the Paracelsus Medical University Salzburg, Salzburg, Austria

## Abstract

Recessive dystrophic epidermolysis bullosa (RDEB) patients suffer from chronic and repeatedly infected wounds predisposing them to the development of aggressive and life-threatening skin cancer in these areas. Vitamin D3 is an often neglected but critical factor for wound healing. Intact skin possesses the entire enzymatic machinery required to produce active 1-alpha,25-dihydroxyvitamin D3 (calcitriol), underscoring its significance to proper skin function. Injury enhances calcitriol production, inducing the expression of calcitriol target genes including the antimicrobial peptide cathelicidin (hCAP18), an essential component of the innate immune system and an important wound healing factor. We found significantly reduced hCAP18 expression in a subset of RDEB keratinocytes which could be restored by calcipotriol treatment. Reduced scratch closure in RDEB cell monolayers was enhanced up to 2-fold by calcipotriol treatment, and the secretome of calcipotriol-treated cells additionally showed increased antimicrobial activity. Calcipotriol exhibited anti-neoplastic effects, suppressing the clonogenicity and proliferation of RDEB tumor cells. The combined wound healing, anti-microbial, and anti-neoplastic effects indicate that calcipotriol may represent a vital therapeutic option for RDEB patients which we could demonstrate in a single-patient observation study.

## Introduction

Epidermolysis bullosa (EB) refers to a group of rare inherited skin disorders characterized by skin fragility, blistering, and erosions following minor trauma. The underlying cause of EB lies within mutations that affect various genes crucial to the structural integrity of the dermoepidermal junction (DEJ)^1^. Recessive dystrophic epidermolysis bullosa (RDEB) is caused by mutations in *COL7A1* which encodes for type VII collagen, the main component of anchoring fibrils that function to attach the epidermis to the underlying dermis^2^. Due to loss of functional type VII collagen, patients with RDEB suffer from chronic open wounds which are susceptible to microbial infections that further delay wound healing and promote ongoing inflammation (as reviewed in^3^). Additionally, >90% of RDEB patients develop an aggressive and life-threatening cutaneous squamous cell carcinoma at sites of chronic and long-term skin wounds, indicating that tumorigenesis is related to the pathology of RDEB^4,5^. Recently, it was demonstrated that innate immune sensing of microbial products promotes wounding- and inflammation-induced skin tumorigenesis^6^, highlighting that topical antimicrobials and local wound care are critically important in wound management and possibly cancer prevention in RDEB. Currently, no general “standard” therapy for the treatment of non-healing and severely infected wounds in RDEB exists, and every patient is treated on an individual basis^7,8^. Existing approaches all come with disadvantages. Antiseptic baths are time-consuming, exhausting, and painful, as all dressings must be carefully removed. Topical sulfonamides containing silver have questionable efficacy and are associated with potential silver toxicities^9,10^, and long-term application of antibiotic and antiseptic ointments risks the emergence of multiresistant bacterial strains^11^. Thus, alternative strategies to manage chronic and infected wounds in RDEB are needed.

Vitamin D3 is a factor that is often overlooked but is critical for proper wound healing and tissue repair. The skin serves as the primary source of vitamin D3 for the entire body. UVB radiation in sunlight triggers the synthesis of cholecalciferol, the inactive pro-form which enters the circulation and undergoes 2 further hydroxylation steps, first in the liver to generate 25-hydroxyvitamin D (25D3 or calcidiol), and finally in the kidneys to generate the active form 1-alpha,25-dihydroxyvitamin D3 (1,25(OH)2D3), also known as 1,25D3 or calcitriol. Of note, while other tissues and organs obtain active VD3 via the circulation, skin keratinocytes are unique in that they possess the entire enzymatic machinery required to produce active calcitriol, independent of renal and hepatic hydroxylation steps^12^. Calcitriol is a potent ligand for the vitamin D receptor (VDR), a transcription factor which mediates most of the physiological actions of this hormone. Keratinocytes also express VDR, enabling them to respond to the calcitriol they produce, and underscoring the importance of this signaling axis to proper skin function. Under homeostatic conditions, the calcitriol/VDR complex modulates the expression of genes involved in keratinocyte proliferation and differentiation, and the maintenance of barrier function^12,13^. Skin injury further enhances production of calcitriol, triggering the expression of VDR-target genes involved in wound healing, most notably the antimicrobial peptide cathelicidin (*CAMP*)^14^. In humans, *CAMP* (also known as hCAP18 or LL-37) is the sole member of the cathelicidin family of antimicrobial peptides (AMPs), evolutionary conserved molecules that form part of the innate immune system and serve as an important first line of defense against infections (as reviewed in^15,16^). hCAP18 is initially expressed as an inactive precursor protein that is processed by serine proteases to the bioactive LL-37 AMP which exhibits direct antibacterial, antiviral, and antifungal activity^17,18^. Additionally, LL-37 exerts other biological activities important for wound healing including modulation of innate and adaptive immune responses, and promoting neovascularization and cellular migration which enhances the re-epithelialization of healing skin^19,20^.

Taken together, vitamin D3 enables keratinocytes to recognize and respond to wounding and infection by enhancing antimicrobial defenses and initiating repair processes. These findings are relevant in the context of RDEB, as limited sun exposure due to wound dressings and reduced outdoor activity of patients could lead to a local vitamin D3 deficiency in the skin^21^. We propose that enhancing active vitamin D3 levels at sites of injury where it is needed could be beneficial to wound healing and control of infections in RDEB patients. Here we provide pre-clinical evidence that *in vitro* treatment of RDEB keratinocytes with the active vitamin D3 analog calcipotriol elicits wound healing effects and enhances local antimicrobial defense. Additionally, calcipotriol exhibited anti-neoplastic effects against RDEB tumor cells, underscoring its potential as a safe therapeutic option for managing non-healing wounds in this patient group.

## Results

### Low doses of calcipotriol do not inhibit cell proliferation of RDEB keratinocytes

**Figure 1:**
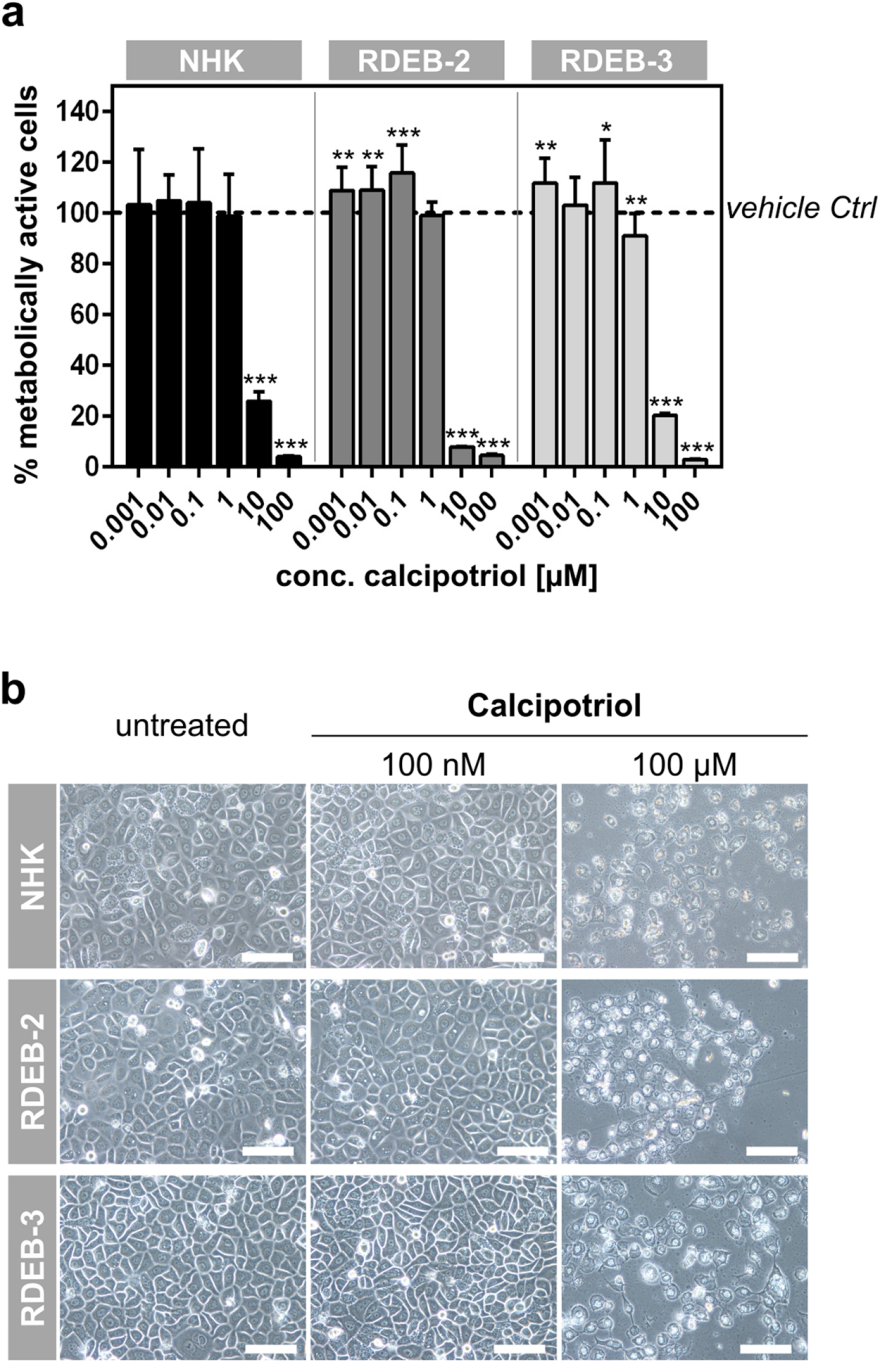
Calcipotriol at low concentrations is not toxic to RDEB keratinocytes. (**a**) Two RDEB-2, RDEB-3, and normal human keratinocyte (NHK) cell lines were treated with increasing concentrations of calcipotriol (0.001–100 µM) for 72 hrs and MTT assays performed to assess cell viability. The percentage of metabolically active cells was calculated relative to DMSO-treated controls. Anti-proliferative effects were observed at concentrations of ≥1 µM calcipotriol. (**b**) Microscopic analysis revealed cell toxicity in all cell lines investigated using 100 µM calcipotriol after 72 hrs, whereas cells treated with 100 nM calcipotriol showed similar cell morphology to untreated controls. Scale bar: 50 µm.

To facilitate the translation of our findings into a clinical therapy for RDEB patients, we performed all our studies with the active vitamin D3 analog calcipotriol, as it represents the active ingredient in a topical ointment already approved and indicated in the treatment of psoriasis^22^. We reasoned that any evidence of efficacy we obtained from our preclinical studies could be used to support the future re-purposing of calcipotriol for off-label use in RDEB. Calcitriol and its analogs are known to inhibit cell proliferation^23^. In the context of psoriasis, calcipotriol at a concentration of ~121 µM (Psorcutan®, 50 µg/g) is used in part to combat the abnormal hyperproliferation of keratinocytes associated with this disorder. As inhibition of cell proliferation would be detrimental to wound healing, we first determined the effective concentration range of calcipotriol to be used in wound healing studies in RDEB. Normal human keratinocytes (NHK) and RDEB keratinocytes were treated for 72 hrs with increasing concentrations of calcipotriol and proliferation was measured by MTT assay. We observed that at the highest concentrations of 10–100 µM, calcipotriol was toxic to keratinocytes (Fig. 1a,b). However, at concentrations up to 1 µM, calcipotriol had no negative impact on cell proliferation. At a concentration of 100 nM, calcipotriol treatment even resulted in a statistically significant albeit small positive impact on proliferation in the two RDEB keratinocyte cell lines tested (Fig. 1a).

### RDEB keratinocytes are responsive to calcipotriol

**Figure 2:**
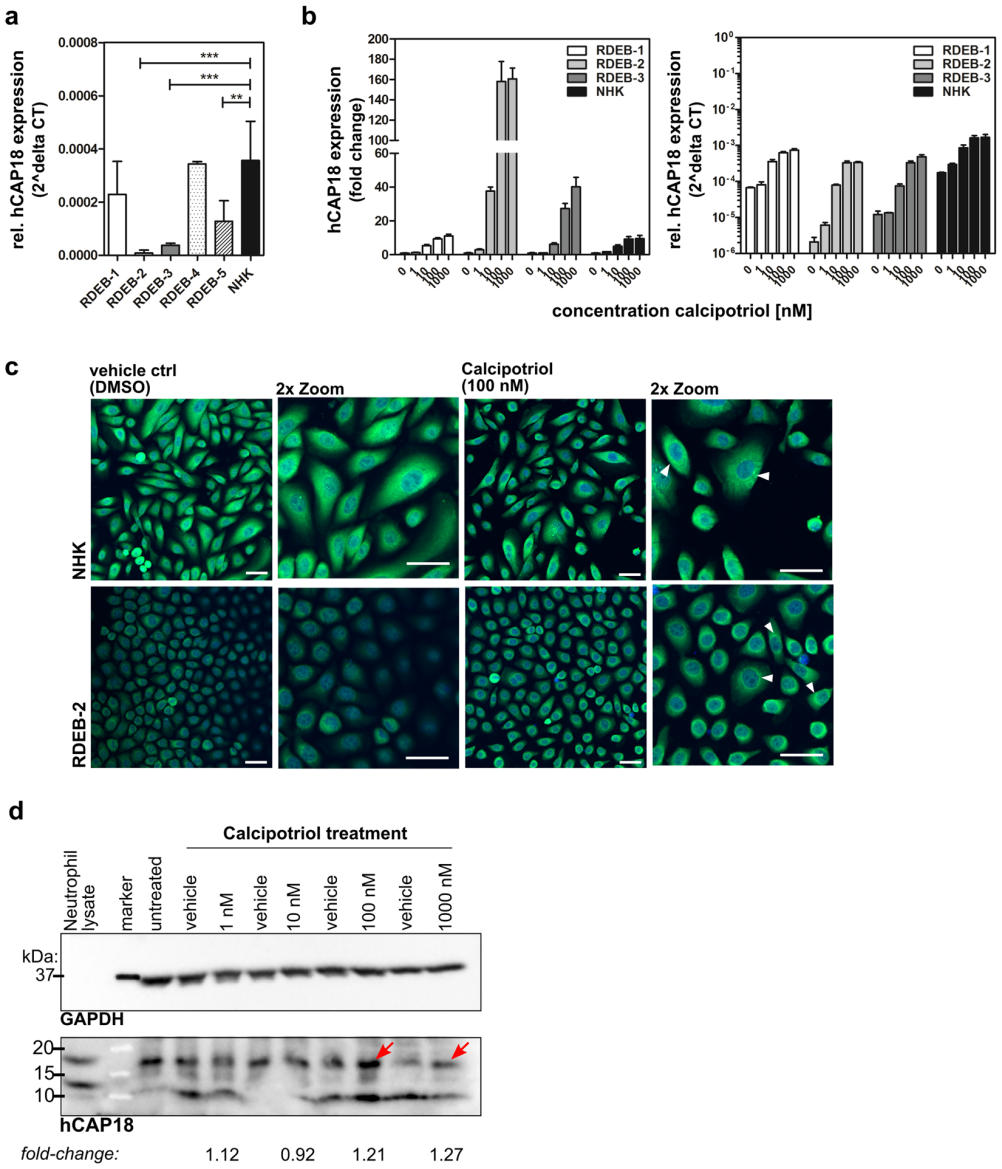
Calcipotriol treatment restores cathelicidin expression in RDEB keratinocytes. (**a**) sqRT-PCR demonstrated significantly reduced hCAP18 mRNA expression in 3 of 5 RDEB keratinocyte lines compared to NHK (unpaired Student’s *t* test, two tailed). (**b**) Concentration-dependent induction of hCAP18 mRNA in RDEB and NHK keratinocytes after 24 hrs calcipotriol treatment. hCAP18 expression was normalized to the housekeeping gene GAPDH. Experiments (n = 2) were carried out in triplicates and mean ± SD is shown. (**c**) Immunofluorescence staining of cathelicidin (green) following 100 nM calcipotriol treatment for 48 hrs. Scale bar = 50 µm; blue = DAPI staining. Perinuclear staining indicated by arrowheads. (**d**) Western blot analysis for cathelicidin (18 kDa) after treating RDEB-3 cells with increasing conc. of calcipotriol compared to DMSO controls. First, the membrane was used to determine cathelicidin expression analysis after which the blot was stripped and reprobed with anti-GAPDH antibody to confirm equal protein loading. After visualization cathelicidin blot was cropped at predicted protein size and as confirmed by the positive control for presentation. The full-length blots are provided in Suppl. Figs S8 and S9. Fold change was calculated using densitometric analysis. Human neutrophil lysate = cathelicidin positive control. Red arrows indicate cathelicidin protein band.

In order to provide evidence that calcipotriol treatment could have a clinical benefit in RDEB, we then investigated the responsiveness of 5 different RDEB keratinocyte cell lines to calcipotriol using induction of cathelicidin (henceforth referred to as hCAP18), a direct VDR-target gene, as a readout. We treated RDEB and NHK cell lines with increasing concentrations of calcipotriol and measured hCAP18 mRNA expression by sqRT-PCR 24 hrs later. Interestingly, we found that basal levels of hCAP18 were significantly lower in 3 out of 5 RDEB cell lines when compared to NHK (Fig. 2a). While calcipotriol treatment induced hCAP18 expression in a concentration-dependent manner in all cell lines tested (Fig. 2b), induction was most robust in RDEB-2 and -3, the two cell lines with the lowest basal hCAP18 expression in our study. In RDEB-2 cells, treatment with 100 nM calcipotriol led to a ~150–fold enhancement of hCAP18 expression (Fig. 2b, left panel), resulting in levels that are comparable to those of normal keratinocytes under basal (untreated) conditions (Fig. 2b, right panel). Treatment of cells with 1000 nM calcipotriol did not result in further significant increase compared to the 100 nM treatment concentration in most cell lines tested. Taken together with the proliferation data above, our results suggest an effective threshold treatment concentration of 100 nM calcipotriol in RDEB.

The increase in hCAP18 mRNA upon calcipotriol treatment translated into enhanced protein levels that could be observed after 48 hrs. RDEB-2 and -3 cells, as well as NHK, were cultured in the absence or presence of 100 nM calcipotriol. After 48 hrs, immunofluorescence and Western blot assays were performed using an antibody against hCAP18 (~18 kDa). Immunofluorescence microscopy revealed a robust induction of hCAP18 in RDEB-2 and -3 cells (Fig. 2c and Suppl. Fig. S1) following calcipotriol treatment. hCAP18 displayed the typical perinuclear localization already described for this protein^24^. Western blot also confirmed the upregulation of hCAP18 protein in response to calcipotriol treatment especially at higher concentrations (Fig. 2d). Thus, RDEB keratinocytes are responsive to calcipotriol treatment, upregulating hCAP18 expression accordingly.

### Calcipotriol enhances wound closure in RDEB *in vitro*

**Figure 3:**
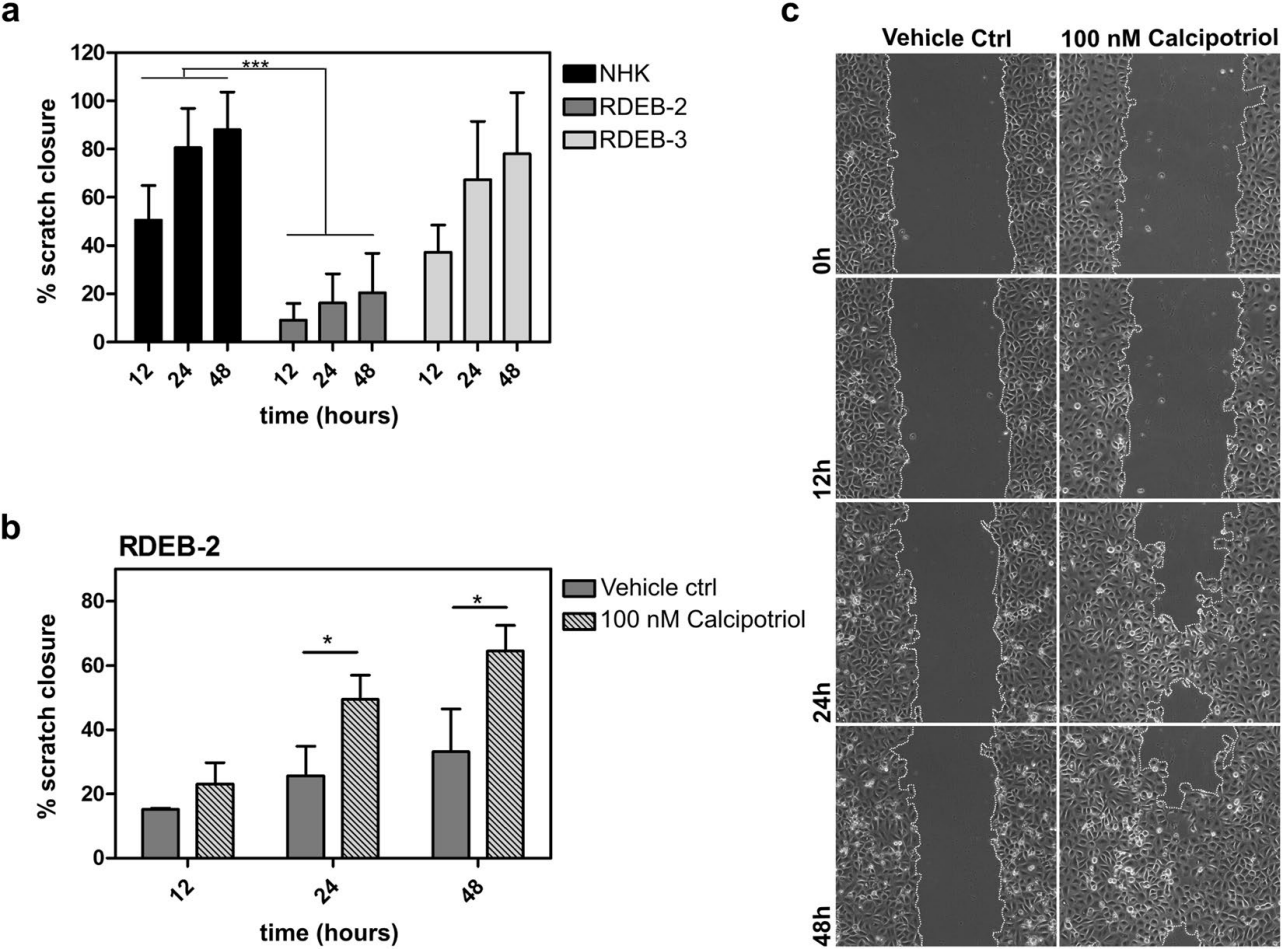
Calcipotriol enhances scratch closure in RDEB monolayers. (**a**) Basal scratch closure of NHK, RDEB-2 and RDEB3 cells were monitored over a 48-hr time period by live microscopy. Experiments were carried out in duplicates and mean ± SD of 6 independent experiments is shown. RDEB-2 keratinocytes showed significantly reduced closure rates compared to NHK (two-way ANOVA repeated measures). (**b**) Calcipotriol treatment of RDEB-2 cells significantly enhanced scratch closure rates by 1.9-fold after 24 and 48 hrs. The mean ± SD of 3 independent experiments is shown (unpaired Student’s t-test, two tailed). (**c**) Microscopic analysis of scratch closure upon treatment of RDEB-2 cells with 100 nM calcipotriol or DMSO at indicated time points. Pictures were taken using a Nikon Eclipse Ti microscope, 10x magnification.

LL-37, the active AMP processed from hCAP18, has been shown to mediate wound healing processes by stimulating re-epithelialization^19^. To evaluate the impact of calcipotriol on wound closure in RDEB we performed *in vitro* scratch assays in confluent monolayers of RDEB-2 and RDEB-3 cells, as these two lines demonstrated significant induction of hCAP18. Scratch closure, in the absence or presence of 100 nM calcipotriol, was assessed by live microscopy over a 48 hr period, and compared to the wild type NHK cell line. NHK typically achieved closure with a median time of about 24 hrs without treatment. Closure rates in untreated RDEB keratinocyte monolayers, however, varied from slightly delayed in RDEB-3 cells to significantly reduced in RDEB-2 keratinocytes. RDEB-3 showed 67.3 and 78% scratch closure after 24 and 48 hrs respectively in the absence of treatment, whereas RDEB-2 cells typically achieved only 16.2 and 20.5% closure at the same time points (Fig. 3a). Treatment with calcipotriol did not significantly enhance scratch closure further in either NHK or RDEB-3 keratinocytes (Suppl. Fig. S2). However, treatment of RDEB-2 keratinocytes with 100 nM calcipotriol, led to significantly enhanced wound closure rates of 1.9-fold over vehicle-treated controls at 24- and 48 hrs (Fig. 3b,c). Although cells were starved of growth factors during the 48-hour observation period, they were not mitotically inactivated prior to making the scratch. Therefore both ongoing cell proliferation and enhanced migration likely contributed to scratch closure in this assay. Importantly, both are physiological processes crucial for proper wound closure.

### Calcipotriol enhances innate immune defense in RDEB

**Figure 4:**
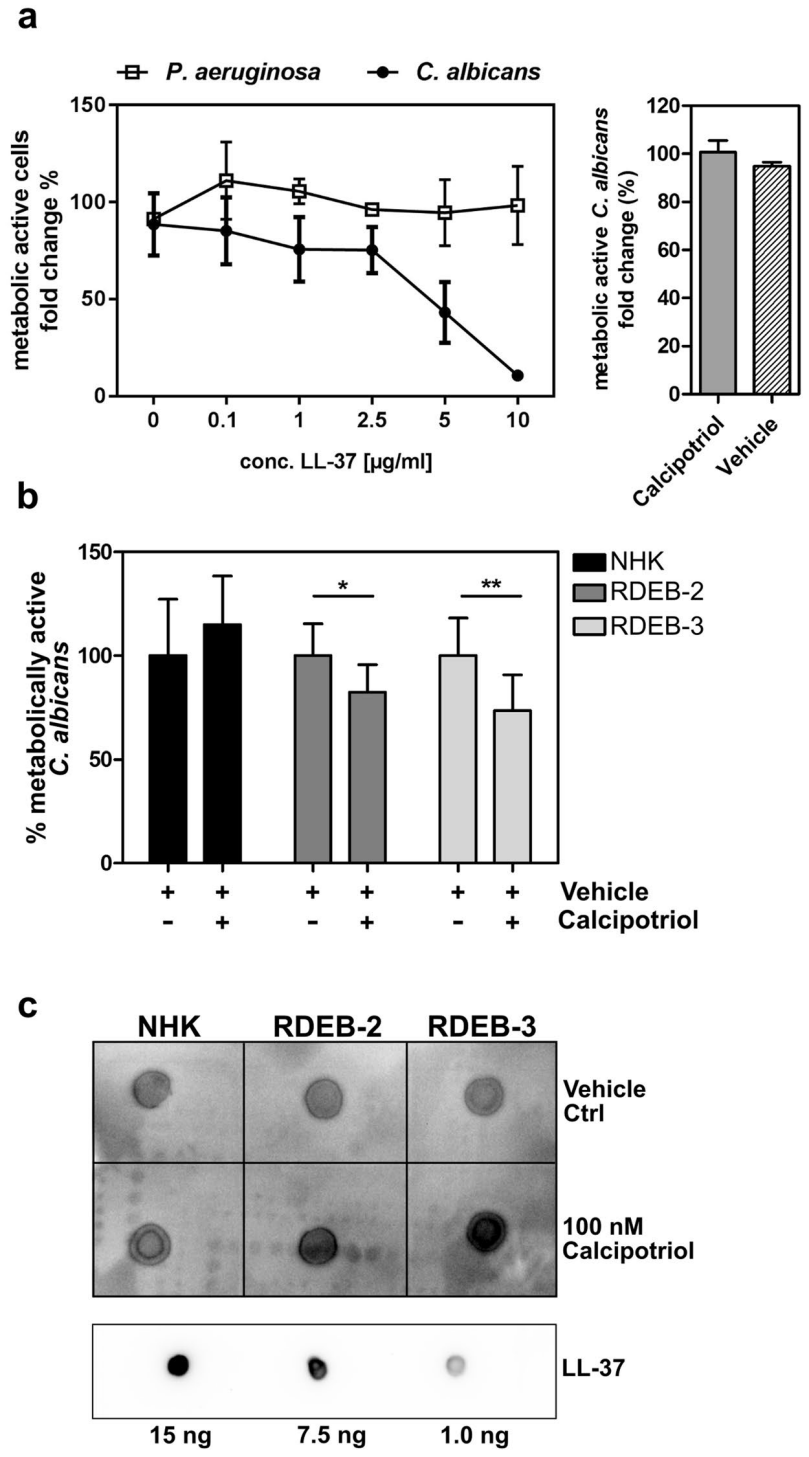
Secretome of calcipotriol-treated RDEB cells exhibit enhanced antimicrobial activity. (**a**) *C. albicans* or *P. aeruginosa* (35,000 CFU early log-phase) were treated with different concentration of LL-37 (0–10 µg/ml) and the number of metabolically active cells was measured in a resazurin-based antimicrobial activity assay. No antiproliferative effect of 100 nM calcipotriol itself was observed when incubated with *C. albicans* (right panel) (**b**) NHK, RDEB-2 and RDEB-3 cells were treated with 100 nM calcipotriol (+) or vehicle control DMSO (−) for 48 hrs, and culture supernatants were harvested and used in a resazurin assay against *C. albicans* as described above. Mean ± SD of five experiments is shown. Statistical analysis was performed as described in the materials and methods section. (**c**) Dot blot assay measuring cathelicidin in concentrated cell culture supernatants of calcipotriol- vs vehicle-treated NHK, RDEB-2 and RDEB-3 cells. Decreasing amounts of spotted LL-37 peptide served as quality control.

RDEB patients are susceptible to wound infections which in turn can hinder the healing process. In this respect, calcipotriol-mediated induction of hCAP18/LL-37 could impart a significant clinical benefit to patients by controlling infection. To investigate this, we established a resazurin-based antimicrobial assay to test the ability of a custom synthesized LL-37 peptide (0.5–10 µg/ml) to inhibit the growth of *P. aeruginosa* or *C. albicans*, both previously described as wound colonizers in EB^25^. Reduction of resazurin to the highly fluorescent resorufin served as an indicator of microbial viability and growth. Fluorescence readings were taken every hour for 24 hrs to evaluate microbial growth kinetics. While the viability of *P. aeruginosa* was unaffected under these assay conditions, inhibition of *C. albicans* by LL-37 was dose dependent and could readily be measured in our assay (Fig. 4a, left panel). We therefore used *C. albicans* as the target for subsequent investigation of the antimicrobial potential of the RDEB cell secretome upon calcipotriol treatment. NHK, RDEB-2, and RDEB-3 keratinocytes were treated for 48 hrs with 100 nM calcipotriol or the vehicle control and supernatants were harvested for co-incubation with *C. albicans* as described above. Notably, we observed reduced microbial growth when *C. albicans* was incubated with conditioned medium from calcipotriol-treated RDEB cells as compared to vehicle-treated cells (Fig. 4b). Incubation of *C. albicans* directly with 100 nM calcipotriol did not have any impact on its growth rate (Fig. 4a right panel). Thus, the observed anti-microbial effects were not directly caused by calcipotriol, but rather by a factor secreted by the treated cells, likely LL-37. In this regard we could confirm the secretion of cathelicidin into the cell culture medium of treated cells which was already detectable at 24 hrs by dot blot assays (Fig. 4c). These results demonstrate that treatment with calcipotriol can enhance the local antimicrobial defense of RDEB keratinocytes.

### Anti-neoplastic effects of calcipotriol in RDEB-SCC

**Figure 5:**
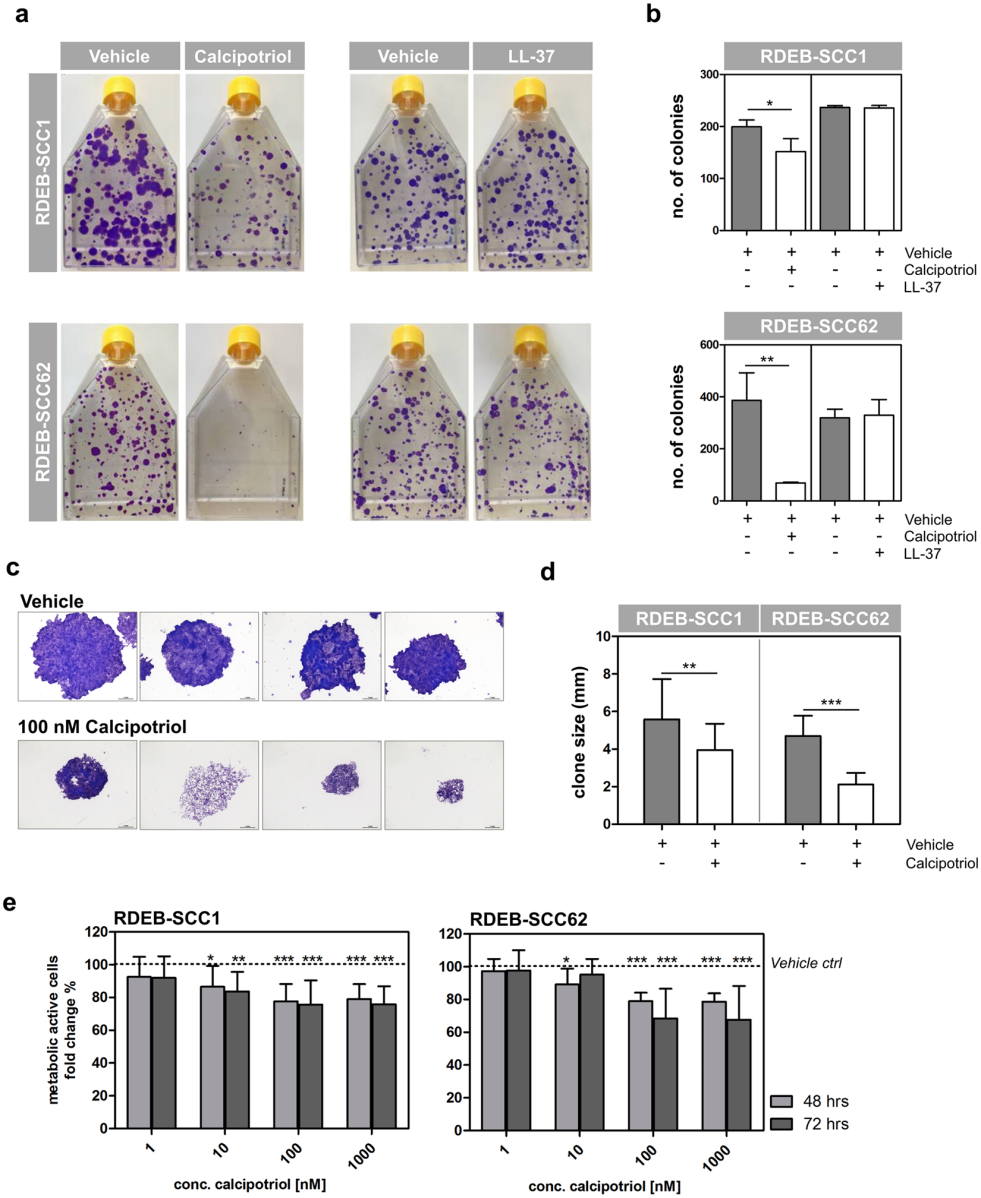
Calcipotriol inhibits clonogenicity of RDEB SCC cells. (**a**) RDEB-SCC cells were plated in T175 flasks (3 cells/cm^2^) and grown in the presence of 100 nM calcipotriol, 500 ng/ml LL-37 peptide or vehicle controls up to 18 days. Colonies were stained with crystal violet. Calcipotriol treatment significantly reduced colony number (**b**) and colony size (**c** + **d**), whereas LL-37 peptide showed no effect. Mean ± SD of three experiments is shown. Scale bar: 1 mm. (**e**) RDEB-SCC cells were cultured in the presence of 1–1000 nM calcipotriol or DMSO and viability assayed by MTT after 48 and 72 hrs. Data are presented as percentage of viable cells, relative to the vehicle treated control cells (100%, dotted line). Mean ± SD of at least 2 experiments performed in quadruplicates is shown. Statistical significance was calculated as described in the materials and methods section.

Vitamin D3 is reported to have anti-neoplastic effects^26^, which is an important consideration for RDEB patients who are prone to developing aggressive cutaneous squamous cell carcinoma (SCC) at sites of chronic wounding. We therefore investigated the effects of calcipotriol treatment on several patient-derived RDEB-SCC cell lines in clonogenicity and proliferation assays. Clonogenicity assays assess the reproductive integrity of cells, i.e. their ability to proliferate indefinitely, and are often used to evaluate the efficacy of chemotherapeutic agents against tumor cells^27^. RDEB-SCC cells, plated at a density of 3 cells per cm^2^ in 175 cm^2^ flasks, were cultured for up to 18 days in the absence or presence of 100 nM calcipotriol. We observed a striking inhibition in the growth of colonies (defined as >50 cells) in 2 tumor lines, RDEB-SCC1, and RDEB-SCC62, upon treatment with calcipotriol. A third SCC line did not form colonies in this assay and was not used further. Treatment with 100 nM calcipotriol resulted in a significant reduction in both number (Fig. 5a,b) and size (Fig. 5c,d) of tumor colonies as compared to vehicle-treated controls. Notably, this anti-neoplastic effect was mediated by calcipotriol independent of LL-37, as direct application of 500 ng/ml LL-37 peptide showed no effect on the clonogenic potential of these tumor cells (Fig. 5a,b).

We additionally verified these results in MTT assays confirming the reduced proliferation of RDEB-SCC cells upon incubation with calcipotriol, which was significant at the treatment concentration of 100 nM (Fig. 5e). Vitamin D’s inhibitory effects on cell proliferation have been partially attributed to cell cycle arrest via upregulation of the cyclin-dependent kinase inhibitor CDKN1A, also known as p21^WAF1/Cip1^^28^. p21 is known primarily for its role in promoting cell cycle arrest, but is also involved in induction of differentiation and senescence^29^. We also investigated the impact of treatment on expression of E-cadherin, also reported to be modulated by the VDR^30–33^, and which mediates cell–extracellular matrix (ECM) and cell–cell interactions that regulate cell fate and differentiation^34^, including cell-cell contact-mediated inhibition of cell proliferation (as reviewed in^35^). After 24 hours of treatment, we observed a modest increase in p21 protein levels by Western blotting, particularly in RDEB-SCC62 cells, which was most sensitive to the anti-neoplastic effects of calcipotriol in our assays (Suppl. Fig. S3a). Immunofluorescence assays demonstrated enhanced membrane expression of E-cadherin in both tumor cell lines after 48 hours treatment with 100 nM calcipotriol (Suppl. Fig. S3b), in keeping with previously published reports in murine SCC^36^.

### Treatment with low-dose calcipotriol ointment demonstrates efficacy in single-patient observation study

**Figure 6:**
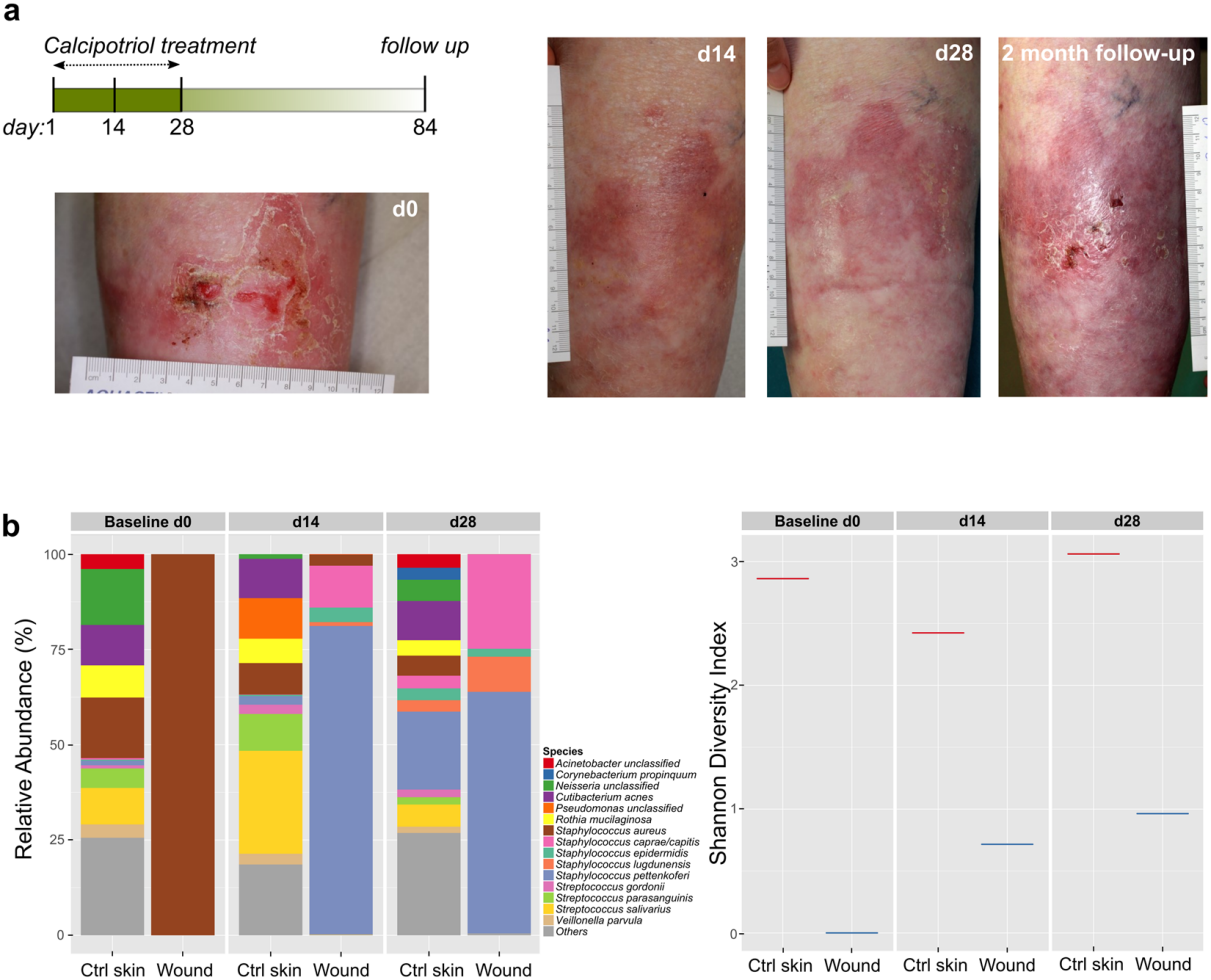
Low-dose calcipotriol ointment – Observation in a single DDEB patient (**a**) A chronic wound on the left lower leg of a 75-year-old DDEB patient was treated with a calcipotriol ointment (0.05 µg/g) over a time period of 4 weeks. Pictures were taken before (d0), during (d14) and at the end of the intervention (d28). A follow-up picture of the treated area was taken 2 months after the last calcipotriol application. (**b**) Relative abundance plot (stacked bar, left panel) and Shannon diversity index (right panel) of microbial species identified in the chronic wound and the corresponding control skin (Ctrl) of the patient in the course of the intervention. Wound microbiota of the DDEB patient revealed less microbial diversity compared to Ctrl skin which could be improved upon calcipotriol ointment application.

In contrast to human and primates, the expression of cathelicidin is not regulated by vitamin D in rodents^14^. The nocturnal life of these animals excludes them from vitamin D photosynthesis^37^. Since we postulated that calcipotriol exerts its antimicrobial and wound healing effects primarily via direct induction of cathelicidin, and because of well-documented species-specific differences in skin wound healing^38^, *in vivo* wound healing studies in mice would not fully recapitulate the human system in this respect, thereby potentially limiting the successful translation of experimental results into clinical application. We therefore performed a single-patient observation study in order to provide a first proof-of-concept as well as investigate potential safety issues of our approach. To this end, a low-dose calcipotriol ointment (0.05 µg/g in Ultraphil®, ~100 nM) was produced by our in-house compounding pharmacy and applied daily, over a period of one month, to a chronic wound of a 75-year-old dominant dystrophic EB (DDEB) patient. Images and wound swabs for microbial analyses were taken prior to treatment and on days 14 and 28 after the start of the intervention (Fig. 6a). We observed complete closure of the wound within the first 2 weeks of treatment, which did not re-open during the remainder of the treatment phase. The patient showed no signs of hypercalcemia as blood calcium levels remained within normal range upon application of the drug (calcium 2.13–2.63 ng/ml), suggesting that application of calcipotriol onto an open wound did not result in significant systemic uptake of the drug. Notably, the patient reported a reduced burden of itch and pain on the wounded area treated with calcipotriol.

Finally, we assessed wound microbiota before and during calcipotrol treatment using shotgun whole-metagenomic sequencing. We observed reduced microbial diversity in the chronic wound compared to corresponding intact skin (Ctrl skin) of the patient. The wound was exclusively colonized with *S. aureus* at the start of intervention (Fig. 6b). This observation corresponds with recent data showing low microbial diversity in RDEB wounds and higher susceptibility to *Staphylococcus* infection in EB patients^39,40^. Notably, treatment with low-dose calcipotriol ointment improved the diversity of the skin microbiota on the affected area already after 14 days, with complete clearance of *S. aureus* by the end of the treatment (day 28).

## Discussion

Our data demonstrate that, in at least a subset of RDEB keratinocytes, treatment with a low concentration (100 nM) of calcipotriol can induce expression of the antimicrobial peptide cathelicidin, enhance wound gap closure, and increase local antimicrobial defense against *C. albicans in vitro*. The observed effects, though significant, were rather small. However, *in vitro* systems cannot fully recapitulate the multi-faceted process of wound healing, especially the contribution of the immune system to this process. Notably, the cathelicidin locally-produced by keratinocytes can serve as a chemoattractant for innate immune cells including neutrophils, which store vast amounts of AMPs^16^, and mast cells, which subsequently produce more LL-37^41^, to the site of injury. Release of AMPs by these supporting cells results in an amplification of the effect, underscoring the utility of this approach in conditions characterized by increased susceptibility to bacterial infections and chronic wounds, such as RDEB. Accordingly, we observed robust wound healing of a long-standing wound of a DDEB patient, along with a reversal of skin microbial dysbiosis, within 2 weeks of treatment with a low-dose calcipotriol ointment. Previous investigations have clearly demonstrated that reduced microbial diversity is associated with disease in various settings, including cancer, autoimmune diseases, inflammatory diseases, and chronic leg ulcers^42,43^. In line with this, we recently showed reduced microbial diversity and an increased abundance of *Staphylococcus species* in RDEB wounds compared to unwounded skin^39^. Amongst this species, *S. aureus* is considered as a pathogenic species known to cause infections in hospitalized patients, impeding the healing process and contributing to the pathophysiology of different diseases^44^. Reduced microbial diversity is accompanied with increased bacterial load of pathogenic microbes (i.e., dysbiosis) which elicits an imbalance of the microbial wound communities driving chronicity and impaired healing process (as reviewed in^45^). In our single-patient observation we demonstrate that calcipotriol application is associated with increased microbial diversity at the wound site and, most notably, the reduction of the pathogen *S. aureus*. Besides *S. aureus* and *C. albicans*, other common wound colonizers in EB include *Streptococcus* and *Pseudomonas* species^25^. Both species have been shown in several studies to be targetable by LL-37^41,46^, pointing to a broad antimicrobial spectrum potentially achievable by this strategy, although this remains to be confirmed in the background of EB.

Vitamin D has also been reported to enhance the expression of human beta defensin 2 (*DEFB2/HBD2*), which belongs to a separate class of antimicrobial peptide, in human keratinocytes, although to a much lower extent as compared to hCAP18^47^. However, we found no evidence of significant differential expression in *DEFB2/HBD2* between NHK and RDEB keratinocytes, and saw only a modest upregulation of *DEFB2/HBD2* upon treatment with calcipotriol *in vitro*, confirming previously published observations (Suppl. Fig. S4). Thus, we ascribe the positive effects on wound healing and antimicrobial defense that we observed in our *in vitro* experiments primarily to the vitamin D3/cathelicidin axis. However, the contribution of other AMPs induced by treatment cannot be ruled out in our single-patient observation study.

Our results also highlight the anti-neoplastic potential of calcipotriol against RDEB tumor cells which is a critical consideration for these patients, given their high risk of developing tumors in areas of chronic wounding. Because of the significant overlap between cellular processes involved in wound healing and tumorigenesis, a major challenge in the field is the development of strategies that will enhance wound healing processes without promoting malignant transformation or vice versa, abrogating tumor progression without inhibiting wound healing. This challenge is underscored by our own clinical experience of the limited applicability of targeted anti-cancer therapies such as the EGFR inhibitor Cetuximab in RDEB-SCC patients. While effective in inhibiting growth of EGFR-positive wt *RAS* RDEB-SCC cells *in vitro* (Suppl. Fig. S5) and in a patient, the debilitating effect on wound healing severely limited its long-term application in this patient (our unpublished observations). In this regard, our observations that calcipotriol could elicit wound healing effects in several RDEB cell lines while inhibiting the outgrowth of single malignant clones is noteworthy and merits further investigation. Our initial investigations demonstrate induction of known tumor suppressors such as p21 and E-cadherin in response to treatment. However, the precise pathways that account for the observed anti-neoplastic effects in RDEB-SCC are not yet completely understood. The pathways modulated by vitamin D/VDR are expected to be manifold, cell type-specific, and will likely be subject to inter-donor variability, requiring comprehensive and integrative –omics approaches coupled with machine-learning data analyses to discern the relevant pathways involved.

An important question raised by our study is whether RDEB patients have a deficiency in vitamin D3 and whether this can be effectively addressed by oral supplementation. Several studies have shown that vitamin D deficiency is prevalent in RDEB patients^21,48^, and that this correlates with low bone mineral density^19^. Data from our clinic confirms that the majority of DEB patients (72%) have insufficient to deficient serum levels of 25(OH)D3 (Suppl. Fig. S6), despite strong recommendation for oral supplementation. This observation is not different from that reported for the general population^49^, but in the context of a disease wherein ongoing tissue repair is needed, such a deficiency is likely to have more pronounced consequences. While systemic oral vitamin D supplementation is critical to address issues of low bone density in patients, we propose that the local tissue concentration of vitamin D is critical for proper skin function. Using hCAP18 as a surrogate marker for activation of vitamin D3-mediated wound healing pathways, we observed reduced levels of hCAP18 mRNA and protein in skin biopsies of some RDEB patients although this did not reach statistical significance (Suppl. Fig. S6). However, as the biopsies were derived from non-wounded skin, these assays do not reflect the situation in an RDEB wound, wherein large sections of the epidermis are lost along with the capacity to actively generate sufficient quantities of calcitriol to support efficient wound healing. Additionally, at least some RDEB keratinocytes may have intrinsic defects in generating calcitriol, severely limiting their ability to respond to injury. For example, in RDEB-2, the most responsive cell line in our study with the lowest basal levels of hCAP18, gene expression profiling identified a significant decrease in the expression of CYP2R1 and CYP27B1, enzymes involved in the generation of calcidiol and calcitriol respectively (Suppl. Fig. S7). Direct topical application of the active metabolite onto chronic wounds where it is critically needed to support local tissue repair would circumvent such deficiencies.

An important aspect of our study is finding the correct concentration of calcipotriol that induces positive wound healing effects in RDEB keratinocytes, which is much lower than the concentration present in the topical ointment used against psoriasis (Psorcutan® 50 µg/g calcipotriol, approximately 121 µM). Based on the data presented here, we have initiated a small clinical trial to assess the efficacy of a low-dose topical application of calcipotriol in improving wound healing in DEB patients. The phase II double-blind, placebo-controlled, randomized, cross-over design study (CALCIDEB2016 Eudranet 2016-001967-35) will address some of the shortcomings of the single-patient observation study presented here, such as the lack of a placebo control, and will enroll both children and adults, thereby allowing for potential correlations to be drawn between age and efficacy of treatment. It could be argued that perhaps direct application of LL-37 might be more effective, as the bulk of vitamin D’s wound healing effects is believed to be mediated via the action of this peptide. A recently concluded Phase I/II trial of hard-to-treat venous leg ulcers demonstrated that treatment with LL-37 (0.5 mg/ml) significantly improved wound healing as compared with placebo^50^. However, several studies show that LL-37 is overexpressed in multiple tumor types, where it is thought to promote tumor cell proliferation, migration, and invasion^51–56^. LL-37 appears to exert contrasting effects, either pro- or anti-tumorigenic, in a tumor/tissue-specific manner^57^. Thus, while the exact nature of the role of LL-37 in the pathophysiology of RDEB-SCC is unknown, we advocate the use of calcipotriol which demonstrated clear anti-neoplastic effects independent of LL-37 at least at the doses examined. Additionally, the known safety profile of calcipotriol facilitates a rapid re-purposing for off-target use in DEB.

While all keratinocyte lines tested responded to calcipotriol by induction of cathelicidin, this effect was robust in only 2 out of 5 lines, with only one of these further showing improvements in all *in vitro* assays shown here. The *in vitro* data suggest that this strategy may benefit only a subset of patients and represents a major limitation of this study. Indeed, in going forward, potential inadequacies in the generation of calcitriol, as well as additional deficiencies in the sensing and response to injury both upstream and downstream of the VDR, warrant closer examination in larger patient cohorts. Likewise, a comprehensive cellular and molecular characterization of what defines a chronic wound will likely contribute to our understanding of what drives defective wound healing in DEB. The integration of data derived from such analyses together with the results from the ongoing clinical trial will provide novel insights that, in the future, will enable the identification of those patients (or wounds) that would benefit most from this treatment strategy.

## Materials and Methods

### Reagents

Calcipotriol (Sigma-Aldrich, St. Louis, MO) was dissolved in DMSO (Sigma-Aldrich). LL-37 peptide [LLGDFFRKSKEKIGKEFKRIVQRIKDFLRNLVPRTES] was ordered from GL Biochem, Shanghai, China and dissolved in DMSO.

### Single patient observation

A 75-year-old dominant dystrophic EB (DDEB) patient (*COL7A1*, c.6127 G > A) volunteered to participate in an observation study involving topical application of a low-dose calcipotriol ointment. The patient intervention was performed with written informed consent at the EB House Austria, Department of Dermatology, with approval of and in accordance with the guidelines of the University Hospital Salzburg and the Declaration of Helsinki (ethical approval number 415-E/2043/9-2016). The wound site to be treated was chosen together with the patient in order to take into account the patient’s needs and wishes. The chosen wound was in an area that was persistently affected in the patient and, as per the patient’s description, had been open for several weeks and was associated with high burden of itch and pain. The low-dose calcipotriol ointment was produced by our in-house pharmacy by diluting Psorcutan® in Ultraphil® to a final concentration of 0.05 µg/g. One gram of ointment was applied to a chronic wound on the lower left leg on a daily basis over a time period of 4 weeks. The patient was advised to apply the calcipotriol ointment onto the wound site without changing their daily wound dressing. Importantly, no other ointment or cream was used during the intervention phase at this site. The patient was asked to come into the clinic every 2 weeks for monitoring and to take pictures of the treated skin area. In addition microbial swabs were taken from wound and corresponding intact skin on the other side of the body at each study visit which served as control (Ctrl). A clinical follow-up was done 2 month after finishing calcipotriol treatment.

### Cell lines and tissue samples

The skin biopsies used in our study were taken under the course of a routine examination at the Department of Dermatology, University Hospital Salzburg. The remaining tissue was used for scientific research after written informed consent of the patients and/or their legal guardians. The Salzburg State Ethics Committee approved the usage of these tissue samples for scientific research (415-EP/73/192-2013). All experiments were conducted in accordance with the Declaration of Helsinki. Details on tissue samples are given in Suppl. Table S1.

Detailed information on cell lines are outlined in Suppl. Table S2. Cell lines RDEB-1,-3 and-5 were generously provided by Prof. Guerrino Meneguzzi, INSERM, Nice, France. RDEB-2 cells have been described previously as RDEB-T4 by Chamorro *et al*.^58^ and were a gift from Prof. Fernando Larcher, CIEMAT-CIBERER, Madrid, Spain. RDEB-SCC1 cells were generously provided by Prof. Leena Bruckner-Tuderman, Freiburg, Germany. RDEB-4, RDEB-SCC62 and NHK cell lines were generated in our own lab using standard isolation techniques. Genomic mutations associated with each cell line were confirmed by standard sequencing techniques. In addition, cell cultures were routinely checked for mycoplasma contamination.

RDEB and normal human keratinocytes (NHK) were maintained in EpiLife® medium (Life Technologies, Carlsbad, CA) supplemented with human keratinocytes growth supplement (Life Technologies) and penicillin/streptomycin where indicated. RDEB-SCC cells were maintained in DMEM/Ham’s F-12 (2:1) (Hyclone) containing 10% serum and growth factors according to the protocol of Rheinwald and Green^59^. All cells were cultured at 37 °C and 5% CO_2_ in a humidified chamber.

### Semi quantitative real-time PCR (sqRT-PCR)

RNA was isolated from cultured cells or tissue samples using the RNeasy Mini Kit or RNeasy Lipid tissue Mini Kit (Qiagen, Hilden, Germany). 1 µg of each RNA sample was treated with DNase I (Sigma-Aldrich) for 20 min at RT. The reaction was stopped by the addition of Stop solution and incubation at 70 °C for 10 minutes. cDNA synthesis was performed using iScript cDNA Synthesis Kit (Bio-Rad, Hercules, CA) according to manufacturer’s protocol. Expression of cathelicidin (hCAP18) and GAPDH was assessed by sqRT-PCR using the IQ SYBR Green Kit (Biorad). Briefly, the reaction consisted of 6.5 µl IQ SYBR Green Supermix 2x (Bio-Rad), 0.5 µL of forward and reverse primer mix (10 µM each) (see Suppl. Table S3 for primer sequences) and 12.5 ng cDNA in a total volume of 13 µl. Each PCR experiment was conducted in duplicates or triplicates and repeated three times or two times, respectively. The PCR reaction was performed in a CFX96 Real Time System C1000 Thermal Cycler (Bio-Rad) under the following conditions: 5 min denaturation phase at 95 °C, followed by 50 cycles with 20 sec denaturation at 95 °C, 20 sec primer annealing at 60 °C, and 20 sec primer extension at 72 °C. To investigate the specificity of the resulting PCR product, an additional melting curve analysis was performed (95 °C for 10 min, 65–95 °C in 0.5 °C increments for 5 sec).

### Western blot and Dot blot analysis

Whole cell protein lysates were prepared by harvesting cells in RIPA Buffer (Santa Cruz Biotechnology, Santa Cruz, CA). Lysates were incubated on ice for 30 minutes followed by a 3 min centrifugation step at maximum speed (≥10.000 × *g*). Supernatants containing soluble proteins were mixed with 4x SDS-PAGE Laemmli sample buffer, heated for 5 minutes at 95 °C and separated on a 4–12% BisTrisNuPage gel (Life technologies) under denaturating conditions for 75 min at 120–150 V. The Precision Plus Protein WesternC Standard (Bio-Rad) was used as molecular weight marker. Proteins were electro-blotted onto a nitrocellulose membrane (Amersham^TM^Hybond^TM^ ECL, GE Healthcare, Little Chalfont, UK) using a wet tank blotting system (Bio-Rad) for 75 min at 250 mA at 4 °C. Unspecific antibody binding sites were blocked with 5% non-fat milk in TBST (10 mM Tris, pH 8.0, 150 mM NaCl, 0.5% Tween 20) for 1 hr, membranes were incubated with antibodies against LL-37 (1:250; Abcam, Cambridge, UK) over night at 4 °C. Membranes were washed three times and incubated with horseradish peroxidase-conjugated anti-rabbit antibodies for 1 hr at room temperature. Blots were washed three times and developed with the Immun-Star WesternC Kit (Bio-Rad) or Immobilon Western Chemiluminescence HRP Substrate (Merck Millipore, Billerica, MA) and visualized with a ChemiDoc XRS Imager (Bio-Rad). Afterwards the blot was stripped to remove antibodies with ReBlot plus mild antibody stripping solution (Millipore, Darmstadt, Germany) for 15 min at room temperature and reprobed using anti-GAPDH antibody (1:10000, DAKO) for 1 hr at room temperature. Subsequent procedures were performed as described above.

For detection of cathelicidin in conditioned medium, 1.5 × 10^5^ cells were seeded in 12 well plates and treated with 100 nM calcipotriol or DMSO for 48 hrs. Protease inhibitors (Complete Mini, Roche, Mannheim, Germany) were added to the harvested supernatants which were then concentrated using Amicon Ultra-0.5 Centrifugal Filter Unit with Ultracel-3 membrane devices (Merck Millipore). The concentrates (3 µl) were spotted onto 0.45 µm nitrocellulose membranes (GE Healthcare) and dried for 1 hr. Membranes were blocked, probed with antibodies, and developed as described above.

### Immunofluorescence analysis

Keratinocytes were seeded in 2-well chamber slides (Thermo Scientific, Braunschweig, Germany) in EpiLife medium without antibiotics and treated with 100 nM calcipotriol or vehicle (DMSO) control for 48 hours. Cells were washed with PBS and fixed with 4% paraformaldehyde for 15 min at room temperature. Cells were washed twice, blocked with 4% BSA in 0.1%Triton X-100/PBS for 1 hour, and then incubated with anti-LL-37 antibody (1:200, Abcam) overnight at 4 °C. Following incubation with goat anti-rabbit Alexa Fluor^®^ 488 (Life Technologies) for 1 hr at room temperature, cells were washed twice with PBS and mounted with SlowFade® Gold Antifade Mountant containing DAPI (Life Technologies). Immunofluorescence images were captured using a confocal laser scanning microscope (Axio Observer Z1 attached to LSM 710, Zeiss, Germany).

### Scratch assay

Keratinocytes were seeded in 12-well plates and grown to confluence. A standardized scratch was made with a 100 µl pipette tip. Cell debris was washed away and EpiLife Medium lacking growth factors and containing either 100 nM calcipotriol or vehicle control (DMSO) was added. Cells were maintained in an Okolab incubation system combined with a Nikon Eclipse Ti microscope fitted with an automatic stage. Scratched areas (3 per well) were photographed every 15 minutes for up to 48 hrs. Images were analysed using NIS elements imaging software AR 4.30.02. The width of the scratch at indicated time points were normalized to t0 values and substracted from 100% to estimate percent wound closure.

### MTT Assay

Cells were seeded into 96-well pates at a density of 5,000 cells/well. The following day, medium containing increasing concentrations of calcipotriol or DMSO was added. Cell viability was assessed after 72 hrs by MTT assay. Briefly, 20 µl of the tetrazolium dye MTT solution (5 g/L) were added to each well and incubated for 2 hours at 37 °C, 5% CO_2_. Cell culture supernatants were removed and cells were lysed by adding 100 µl of DMSO/glycine (6 vol DMSO + 1 vol 0.1 M glycine/NaOH, pH 10.2). Reduction of MTT to formazan was measured at 492 nm/620 nm using a plate photometer (Spark 10 M, Tecan). Each experiment was carried out at least in triplicates.

### Antimicrobial activity

Keratinocytes were cultured in 12-well plates for 48 hrs in EpiLife medium supplemented with either 100 nM calcipotriol or DMSO and without antibiotics. *C. albicans* or *P. aeruginosa* (patient isolate) were grown to early log phase in Luria broth (LB). 35,000 colony forming units/well were seeded into a 96-well black microplate (Greiner Bio-One, Kremsmünster, Austria) and 100 µl of conditioned cell culture medium from untreated or treated cells were added. After 3 hrs incubation at 37 °C, 100 µl of resazurin solution (0.5 nM Resazurin, Sigma-Aldrich) was added. Plates were incubated at 37 °C overnight in a Spark 10 M multiplate reader (Tecan, Grödig, Austria) which measured fluorescence at Ex 535 nm/Em 590 nm every hour. *C. albicans* and *P. aeruginosa* were treated directly with increasing concentrations of LL-37, 100 nM calcipotriol, or DMSO served as controls.

### Clonogenicity assay

RDEB-SCC cells were plated at a density of 3 cells/cm² in T175 cell culture flasks. The next day the medium was replaced with medium supplemented with either 100nnM calcipotriol or DMSO. Fresh medium was added weekly and after an incubation period of up to 18 days cells were washed with PBS, fixed with 4% paraformaldehyde, and stained with 0.05% crystal violet. Following several washes with water, the flasks were air-dried and photographed. Additionally, individual clones were photographed using a Nikon SMZ 745T.

### DNA extraction and metagenomic sequencing

DNA was extracted from swabs that were transferred into Lysing Matrix E tubes (MP Biomedicals) with 500 μl of ATL Buffer (Qiagen). Samples were subjected to bead-beating in a FastPrep-24 Instrument (MP Biomedicals) at 6.0 m/s for 40 s and then centrifuged at 16,000 × g for 5 min. 200 μL of the supernatant were treated with Proteinase K (Qiagen) and incubated at 56 °C for 15 min before extraction in the EZ1 Advanced XL Instrument (Qiagen) using the EZ1 DNA Tissue Kit (Qiagen) according to the manufacturer’s protocol. DNA concentration was quantified using a Qubit fluorometer and the Qubit dsDNA HS Assay Kit (Life Technologies).

DNA libraries were constructed with 50 μL of extracted DNA after shearing by Adaptive Focused Acoustics™ technology (Covaris) using the GeneRead DNA Library I Core Kit (Qiagen). Custom index primers were used for the enrichment of DNA libraries with 14 cycles of PCR. Libraries were quantified using the Agilent DNA 1000 Kit (Agilent Technologies) on an Agilent 2100 Bioanalyzer (Agilent Technologies), normalized and pooled in equimolar concentrations for 101 bp paired-end sequencing on an Illumina HiSeq2000 Instrument at the Next Generation Sequencing Platform (Genome Institute of Singapore).

### Bioinformatics analysis

Demultiplexed reads were subjected to the following preprocessing steps: sequencing bases with quality score <3 were trimmed, and reads <30 bp in length were removed from the analysis using FAMAS (https://github.com/andreas-wilm/famas). Subsequent reads were mapped to the human reference genome (GRCh38) using BWA-MEM^60^ (version 0.7.10-r789) and human host reads were removed from further analysis. MetaPhlAn v2.0^61^ with default parameters was used to profile the microbial composition at each taxonomic level. Shannon diversity indices were calculated using the vegan package in R (version 2.4–5) and all plots were generated with ggplot2 in R.

### Nucleotide sequence accession numbers

Metagenome data have been submitted to NCBI Sequence Read Archive and are accessible through BioProject ID PRJNA488682.

### Statistical analysis

All statistical analyses were performed using GraphPad Prism 5.0 Software. Data were analyzed for normal distribution using Kolmogorov-Smirnov test, D’Agostino & Pearson omnibus normality test and Shapiro-Wilk normality test. Where the data in the two groups to be compared were normally distributed, the unpaired t-test (two-tailed) was used. Where the data to be compared were not normally distributed, the Mann-Whitney test (two-tailed) was used. Results were considered significant when P ≤ 0.05; *P ≤ 0.05, **P ≤ 0.01, ***P ≤ 0.001.

## Electronic supplementary material

Supplementary Information
