# Supplementary material for: Low-dose calcipotriol can elicit wound closure, anti-microbial, and anti-neoplastic effects in epidermolysis bullosa keratinocytes
Source: Sci Rep. 2018 Sep 7;8:13430. doi: 10.1038/s41598-018-31823-6 (PMC6128832; doi:10.1038/s41598-018-31823-6)
Supplement: Supplementary file 1 — Supplementary Information [file 41598_2018_31823_MOESM1_ESM.pdf]

SUPPLEMENTARY INFORMATION

Low-dose calcipotriol can elicit wound closure, anti-microbial, and anti-neoplastic effects in epidermolysis bullosa keratinocytes

Christina Guttmann-Gruber[1]*, Birgit Tockner[1], Cornelia Scharler[2], Clemens Hüttner[1], John E. Common[3,4], Angeline S. L. Tay[3], Simon L. I. J. Denil[3], Alfred Klausegger[1], Andrea Trost[5], Jenny Breitenbach[1], Peter Schnitzhofer[1], Peter Hofbauer[6], Martin Wolkersdorfer[6], Anja Diem[1], Martin Laimer[7], Dirk Strunk[2], Johann W. Bauer[7], Julia Reichelt[1], Roland Lang[7†] and Josefina Piñón Hofbauer[1†]

[1]EB House Austria, Research Program for Molecular Therapy of Genodermatoses, Department of Dermatology, University Hospital of the Paracelsus Medical University (PMU), Salzburg, Austria.

[2]Experimental & Clinical Cell Therapy Institute, Spinal Cord Injury and Tissue Regeneration Center Salzburg (SCI-TReCS), PMU Salzburg, Austria

[3]Institute of Medical Biology, A*STAR, 8A Biomedical Grove, Immunos #06-08, Singapore 138648, Singapore

[4]Skin Research Institute of Singapore, A*STAR, 8A Biomedical Grove, Immunos #06-06, Singapore

[5]University Clinic of Ophthalmology and Optometry, Research Program for Ophthalmology and Glaucoma Research, Paracelsus Medical University Salzburg, Austria

[6] Landesapotheke Salzburg, Department of Production, Hospital Pharmacy, Salzburg, Austria.

[7]Department of Dermatology, University Hospital Salzburg, Paracelsus Medical University

Salzburg, Austria

Table S1. Tissue samples used in hCAP18 mRNA expression analyses

Patient #	Mutation in *COL7A1*	Exon	Sex	Body site
HD #1	-		m	abdomen
HD #2	-		f	inguinal/groin
HD #3	-		m	split skin upper arm
HD #4	-		f	face
HD #5	-		f	axilla
HD #6	-		m	axilla
HD #7	-		m	foreskin
HD #8	-		f	axilla
RDEB #1	c.425A>G, (p.K142R) / c.5261dupC	3 / 59	m	upper arm
RDEB #2	c.8440C>T, (p.R2814X) / c.8440C>T, (p.R2814X)	114 / 114	m	ancle
RDEB #3	c.2005C>T, (p.R669X) / c.2005C>T, (p.R669X)	15 / 15	f	split skin from thigh
RDEB #4	c.976+4A>C / c.976+4A>C	7(i) / 7(i)	m	not known
RDEB #5	c.4048-1G>T / c.8440C>T, (p.R2814X)	34(i) / 114	m	leg

HD = healthy donor; i = Intron

Table S2. Patient-derived cell lines

Cell line	Patient mutation in *COL7A1*	Exon	Immortalization route	Reference
NHK	wild-type	-	E6/E7	Sun et al. 2017[1]
RDEB-1	c.7012C>T, (p.R2338X) / c.7012C>T, (p.R2338X)	90 / 90	E6/E7	Atanasova et al. 2017[2]
RDEB-2	c.6527insC / c.6527insC	80 / 80	SV40	Chamorro et al.2013[3]
RDEB-3	c.1732C>T, (p.R578X) / c.7786delG	13 / 104	E6/E7	Murauer et al. 2010[4]
RDEB-4	c.2858-2859delAG / c.2858-2859delAG	22 / 22	E6/E7	Sun et al. 2017[1]
RDEB-5	c.2828C>T, (p. R2610X) / c.2828C>T, (p.R2610X)	105 / 105	E6/E7	Atanasova et al. 2017[2]

Cell line	Patient mutation in *COL7A1*	Exon	Sex	Age	Primary tumor site	Histology	Reference
RDEB-SCC1	c.8244dupC / c.8244dupC,	111 / 111	F	32	shoulder	N/A	Watt et al. 2011[5]
RDEB-SCC62	c.682+1G>A / c.7474C>T, (p.R2492X)	Intron 5/ 98	F	28	lower arm	Highly differentiated	Sun et al. 2017[1]

Table S3. sqRT-PCR primer sequences

Gene	Orientation	Sequence (5' → 3')	Tm (°C)
hCAP18	fwd	AGG ATT GTG ACT TCA AGA AGG ACG	60
	rev	GTT TAT TTC TCA GAG CCC AGA AGC	60
DEFB2/HBD2	fwd	TGA TGC CTC TTC CAG GTG TTT	59.5
	rev	GGA TGA CAT ATG GCT CCA CTC TT	62
GAPDH	fwd	GCC AAC GTG TCA GTG GTG GA	65
	rev	CAC CAC CCT GTT GCT GTA GCC	65

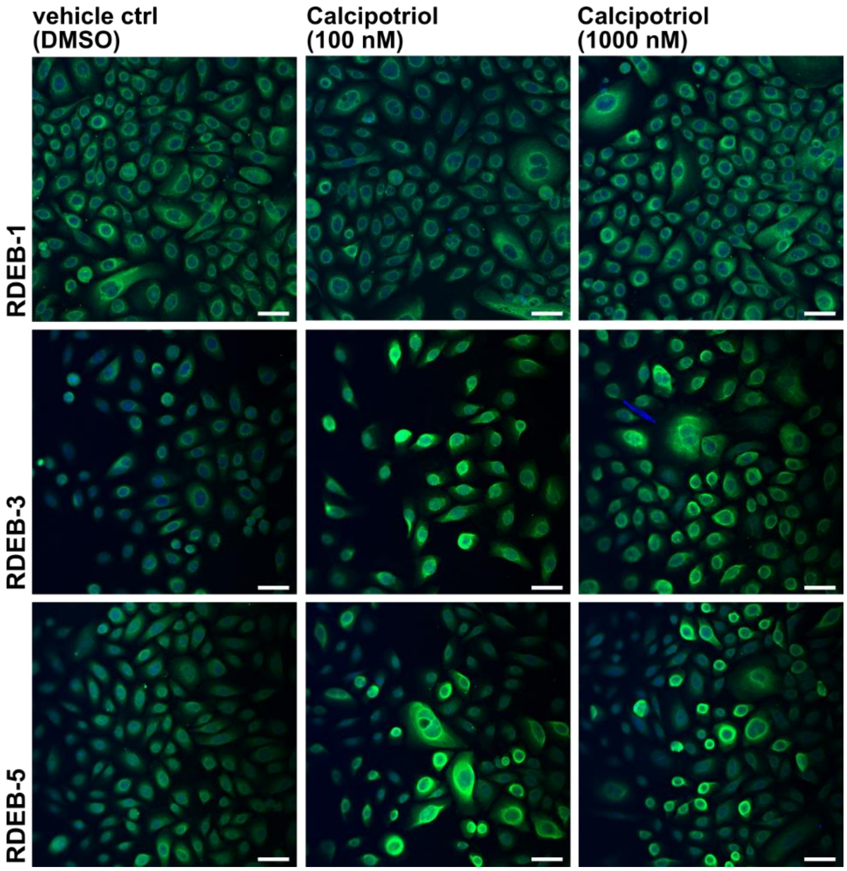

Suppl. Figure S1. Calcipotriol induces expression of cathelicidin as shown by immunofluorescence analysis. RDEB-1, RDEB-3 and RDEB-5 keatinocytes were incubated with 100 nM or 1000 nM calcipotriol or the vehicle control over a time period of 48hrs. Induction of cathelicidin expression was induced in RDEB cells that showed lowest basline expression (RDEB-3, RDEB-5). Cathelicidin (green), DAPI (blue); scale bar: 50 µM

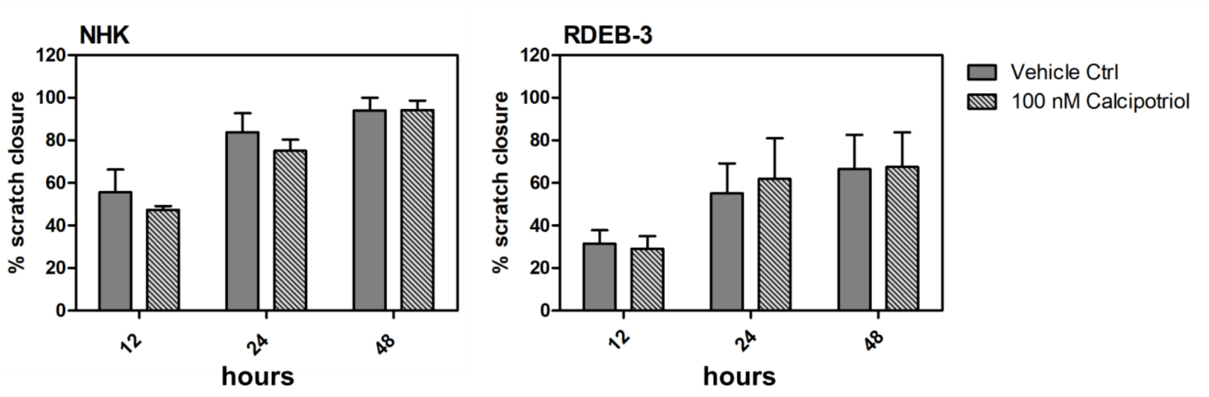

Suppl. Figure S2. Scratch closure in NHK and RDEB-3 keratinocyte monolayers. Cells were treated with 100 nM calcipotriol or the vehicle control over a time period of 48 hours. % scratch closure is presented. The mean ± SEM of 3 independent experiments is shown.

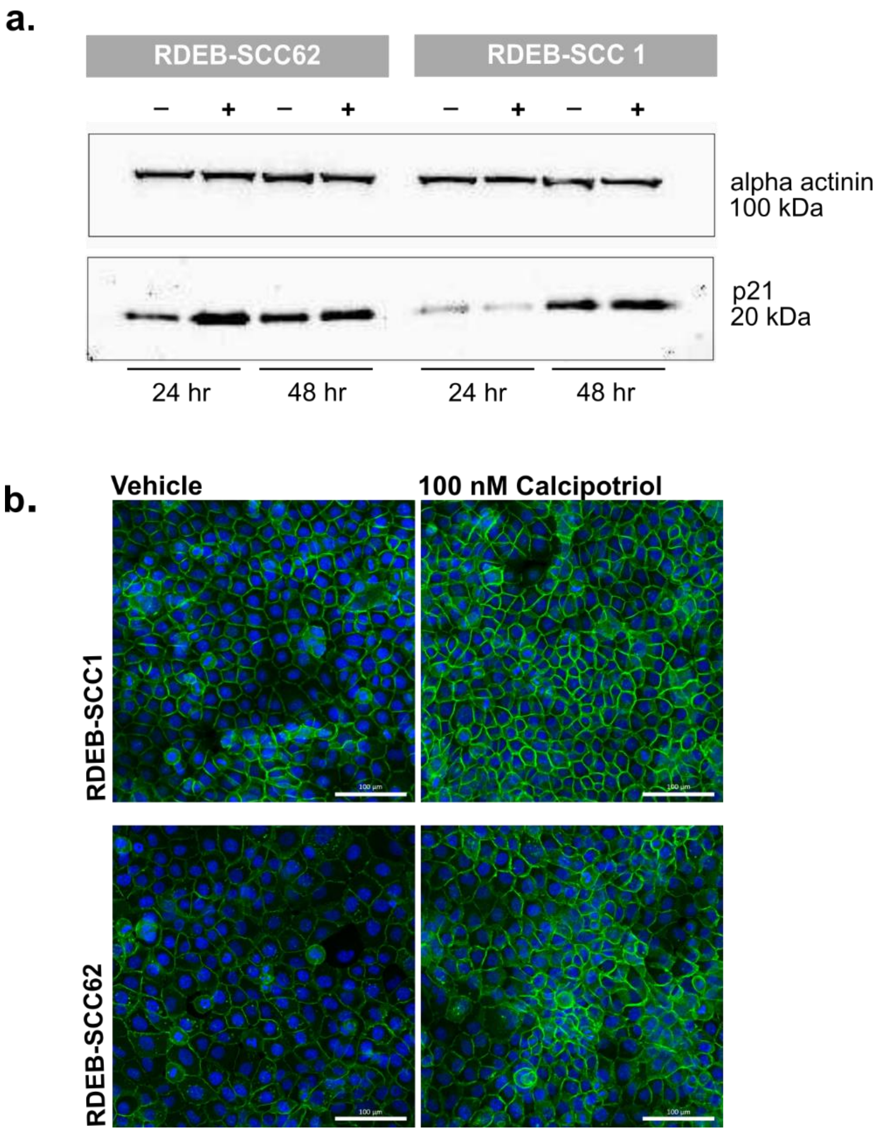

Suppl. Figure S3. Calcipotriol exhibits anti-neoplastic effects on tumor cells. (a) Western blot analysis revealed slightly elevated levels of p21 in RDEB-SCC62, but not in RDEB-SCC1 cells after 24 and 48 hrs calcipotriol treatment. [+] 100 nM calcipotriol; [-] vehicle/DMSO control. Alpha actinin (100 kDa) served as qualitative and quantitative loading control. The full-length blots are provided in Suppl. Fig. S10 (c) Immunofluorescence analysis of RDEB-SCC1 and RDEB-SCC62 monolayers stained for E-cadherin (green) and nuclei (blue; DAPI) after incubation with 100 nM calcipotriol or vehicle control (DMSO). Scale bar: 100 µm.

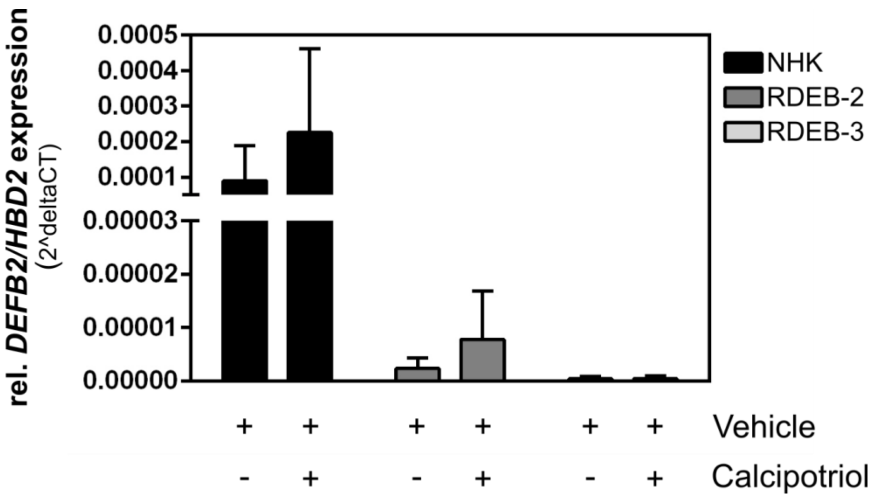

Suppl. Figure S4. Effect of calcipotriol on *DEFB2/HBD2* expression in RDEB keratinocytes. NHK, RDEB-2 or RDEB-3 cells were treated with 100 nM calcipotriol or the vehicle control (DMSO) for 24 hrs and relative mRNA transcript levels were assessed by sqRT-PCR. The DEFB2/HBD2 primers were previously described by Gambichler *et al.*, 2006[6]. Grubbs test was performed to exclude outliers prior to statistical analyses using Mann-Whitney U test (NHK DMSO vs Calcipotriol, P = 0.5429; RDEB-2 DMSO vs Calcipotriol, P> 0.9999; RDEB-3 DMSO vs Calcipotriol, P = 0.6571). Experiments were carried out in duplicates and mean ± SD of two experiments is shown.

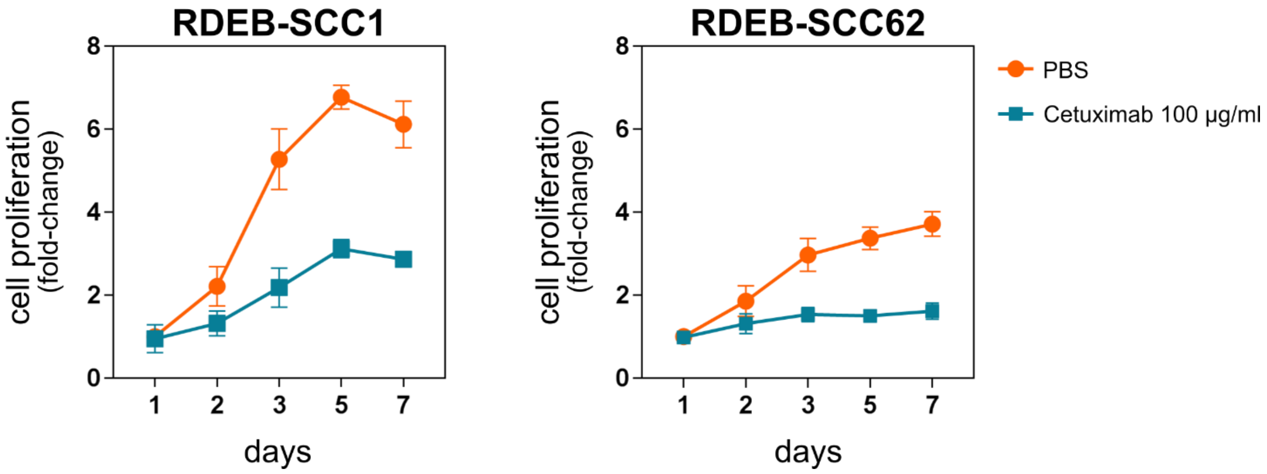

Suppl. Figure S5. Growth inhibitory effects of EGFR inhibitor Cetuximab in RDEB-SCC cells. Two different RDEB-SCC cell lines (RDEB-SCC1 and RDEB-SCC62) were treated with 100 µg/ml Cetuximab over a time period of 7 days. MTT assay revealed striking reduction of cell proliferation already after 3 days treatment compared to the PBS control. Data are presented as mean ± SD of 3 different experiments.

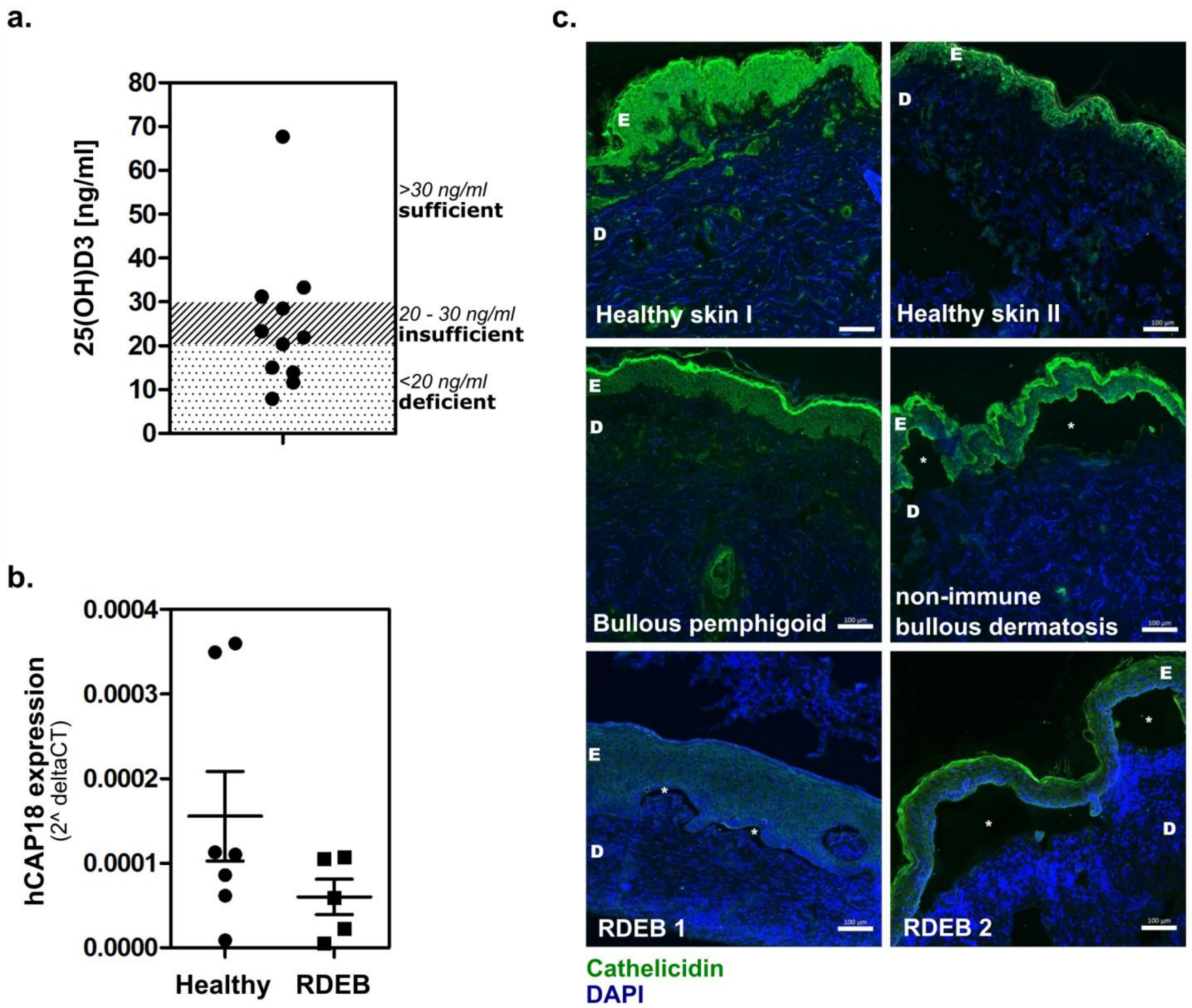

Suppl. Figure S6. 25(OH)D3 and cathelicidin levels in RDEB patients (a) Serum vitamin D levels in DEB patients (RDEB n=10; DDEB n=1). The patient cohort consisted of 5 female and 6 male and had a mean age of 21.5 years. Levels > 30 ng/ml are considered as sufficient, 20 – 30 ng/ml as insufficient and < 20 ng/ml as deficient supply with vitamin D.(b) sqRT-PCR showing transcript levels of hCAP18 in RDEB skin tissue compared to healthy controls. Grubbs test was performed to exclude outliers before statistical significance was calculated using Mann-Whitney U test (*P=0.1490*). (c) Immunofluorescence staining of cathelicidin (green) in various skin tissues including healthy controls, bullous skin diseases and RDEB skin. DAPI counter staining of nuclei. E = epidermis; D = dermis; blisters are indicated with asterisks. Scale bar: 100 µm.

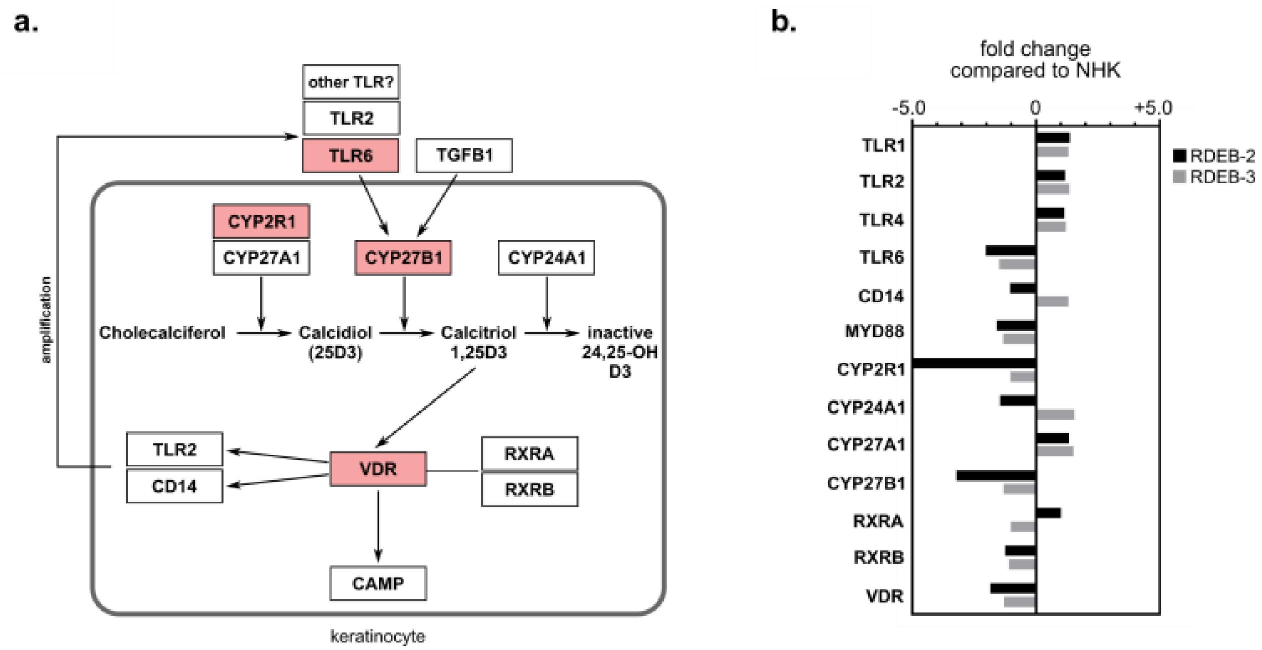

Suppl. Figure S7. Gene expression profiling of RDEB-2 and RDEB-3 compared to NHK. (a) Schematic diagram showing vitamin D metabolism and signaling in keratinocytes. (b) Microarray analysis revealed reduced mRNA expression of CYP2R1 and CYP27B1 expression in RDEB-2 cells compared to NHK, suggesting an intrinsic vitamin D synthesis defect for this cell line.

Abbreviatons: CAMP, human cathelicidin; CD14, cluster of differentiation 14; CYP2R1; 25-hydroxylase; CYP24A1, 24-hydroxylase; CYP27A1, sterol 27-hydroxylase; CYP27B1, 1α-hydroxylase; MYD88, myeloid differentiation primary response 88; RXR, retinoid X receptor; TGFB1, transforming growth-factor beta; TLR, toll-like receptor; VDR, vitamin D receptor;

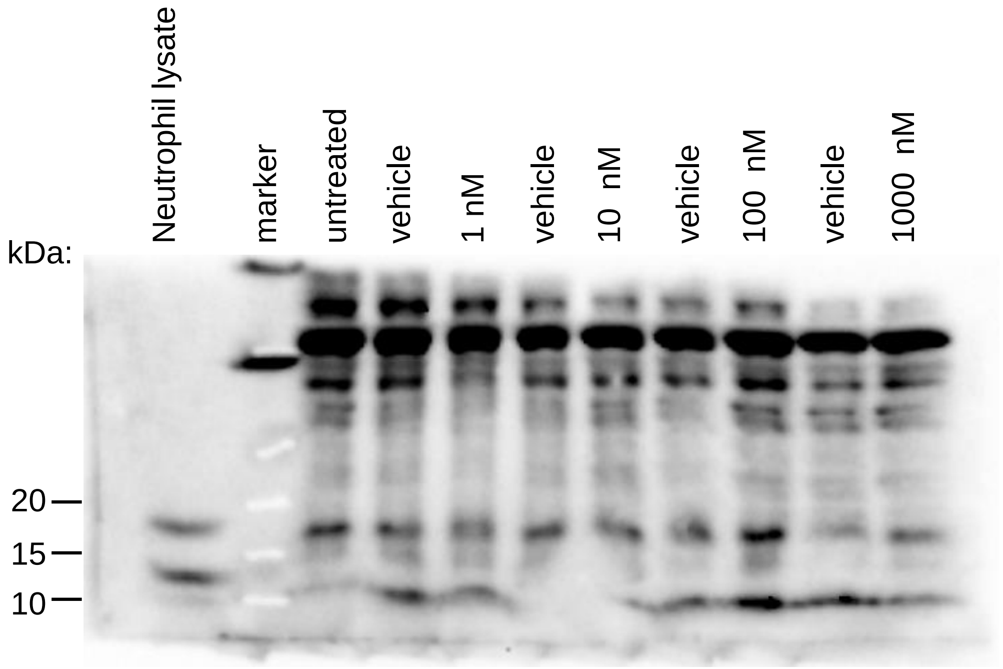

Suppl. Figure S8. Uncropped, full length Western blot showing cathelicidin (~ 18 kDa) expression upon calcipotriol or DMSO (vehicle) treatment. Human neutrophil lysate served as positive control.

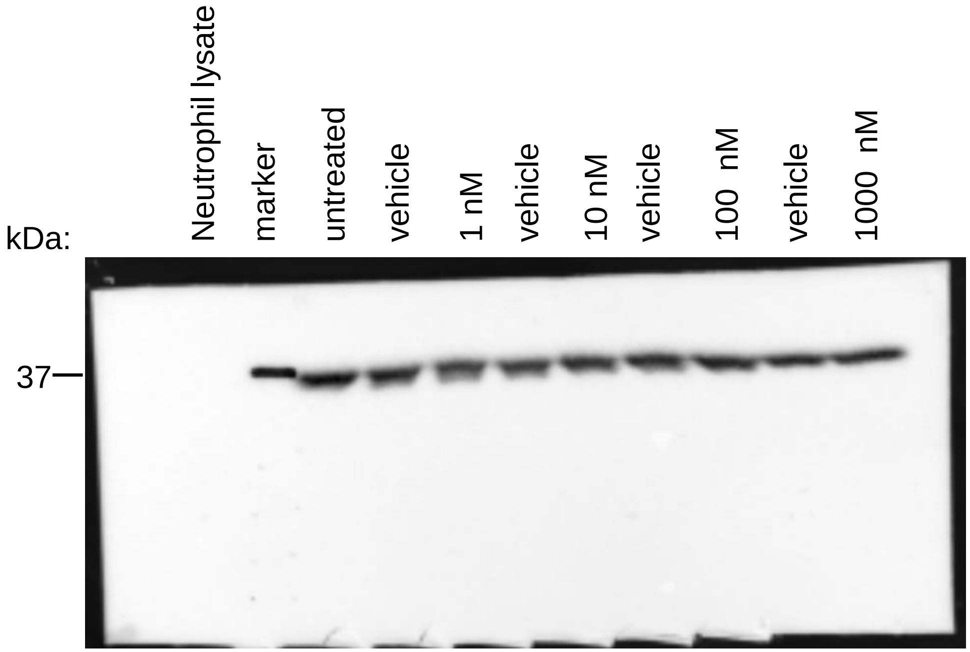

Suppl. Figure S9. Uncropped, full length Western blot showing GAPDH expression (25kDa).

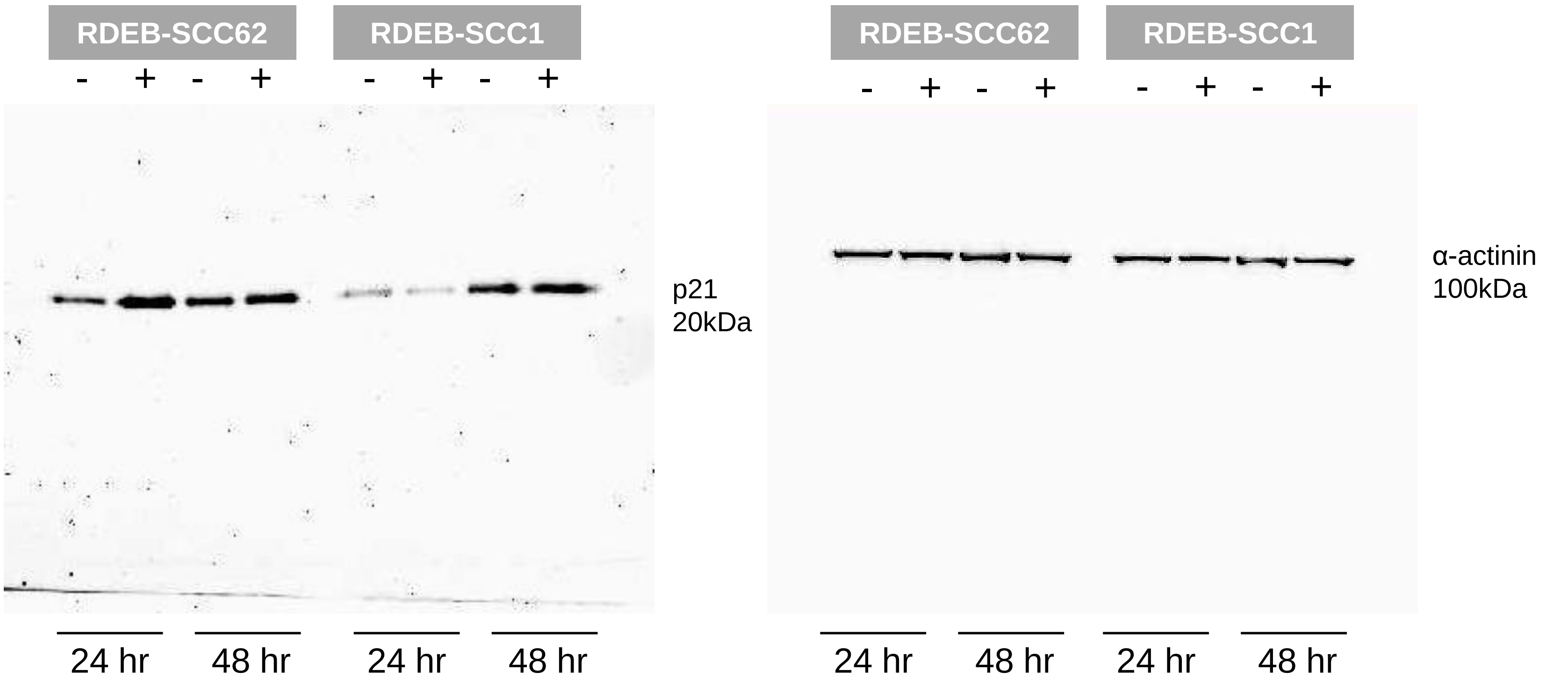

Suppl. Figure S10. Uncropped, full length Western blots showing p21 (21 kDa, left panel) and alpha actinin (100kDa, right panel) expression in RDEB-SCC62 and RDEB-SCC1 cells after 24 and 48 hrs calcipotriol treatment
